# FutureStreams, a global dataset of future streamflow and water temperature

**DOI:** 10.1038/s41597-022-01410-6

**Published:** 2022-06-15

**Authors:** Joyce Bosmans, Niko Wanders, Marc F. P. Bierkens, Mark A. J. Huijbregts, Aafke M. Schipper, Valerio Barbarossa

**Affiliations:** 1 Department of Environmental Science, Radboud University, Heyendaalseweg 135, 6525 AJNijmegen, The Netherlands; 2 Department of Physical Geography, Utrecht University, Princetonlaan 8a, 3584 CB Utrecht, The Netherlands; 3 Deltares, Unit Subsurface and Groundwater Systems, Daltonlaan 600, 3584 BK Utrecht, The Netherlands; 4 PBL Netherlands Environmental Assessment Agency, Bezuidenhoutseweg 30, 2594 AV Den Haag, The Netherlands; 5 Institute of Environmental Sciences (CML), Leiden University, Einsteinweg 2, 2333 CC Leiden, The Netherlands

**Keywords:** Hydrology, Ecology

## Abstract

There is growing evidence that climate change impacts ecosystems and socio-economic activities in freshwater environments. Consistent global data of projected streamflow and water temperature are key to global impact assessments, but such a dataset is currently lacking. Here we present FutureStreams, the first global dataset of projected future streamflow and water temperature for multiple climate scenarios (up to 2099) gridded at a 5 arcminute spatial resolution (~10 km at the equator), including recent past data (1976–2005) for comparison. We generated the data using global hydrological and water temperature models (PCR-GLOBWB, DynWat) forced with climate data from five general circulation models. We included four representative concentration pathways to cover multiple future greenhouse gas emission trajectories and associated changes in climate. Our dataset includes weekly streamflow and water temperature for each year as well as a set of derived indicators that are particularly relevant from an ecological perspective. FutureStreams provides a crucial starting point for large-scale assessments of the implications of changes in streamflow and water temperature for society and freshwater ecosystems.

## Abstract (summary)

**Table:** 

Measurement(s)	Water temperature • Streamflow
Technology Type(s)	water temperature model • hydrological model
Factor Type(s)	climate
Sample Characteristic - Environment	stream
Sample Characteristic - Location	Global

## Background & Summary

Freshwater ecosystems are hotspots of biodiversity that provide vital resources to humanity. These systems are however increasingly threatened by a multitude of anthropogenic pressures, including land cover change, pollution and hydraulic engineering schemes^1–3^. On top of these, climate change will pose a progressively larger threat in the future^2,4^. Ongoing and expected increases in air temperature and changing precipitation patterns alter water temperatures and flow regimes, with water scarcity issues and ecological impacts becoming apparent already at relatively moderate global mean warming levels of 1.5 to 2 °C^4–7^.

Developing effective strategies to alleviate the pressures on freshwater ecosystems worldwide requires globally consistent datasets that can be used to diagnose the threats^1,8^. During recent years, there have been various efforts to create compilations of relevant characteristics of freshwater systems and their surrounding watersheds^8–10^. These efforts have significantly advanced the availability of consistent high-resolution data on for example river network topology, watershed boundaries, streamflow and a variety of catchment characteristics including current climate, geology and soil, and landcover. However, a consistent global dataset that can be used to assess threats to freshwater systems imposed by climate change is yet lacking. Existing global projections of climate-related changes in hydrology typically focus on water quantity only^4,5,11^, without considering water temperature. However, water temperature plays a crucial role in many physical, chemical, and biological processes, including the solubility of oxygen and the performance of aquatic plants and animals^7,12,13^. Thus, consistent data on potential streamflow and water temperature would be an important asset for a plethora of socio-economic as well as ecological analyses, including assessments of potential changes in freshwater biodiversity^7,14^ and the availability of global water resources, in terms of both quantity and quality^15–18^.

Recent developments in models and computational efficiency have enabled us to simulate both streamflow and water temperature consistently, across the globe, and under a range of potential future climate conditions at high spatial and temporal resolution. Our FutureStreams dataset contains weekly streamflow and water temperature at a 5 arcminute resolution (approximately 10 km at the equator) and global extent for multiple climate scenarios up to the year 2099 (see Table 1). For comparison, we also provide streamflow and water temperature data for the recent past (1976–2005). We furthermore include a set of derived streamflow and water temperature metrics that are expected to be particularly relevant from an ecological perspective (see Tables 2 and 3), designed based on indicators of hydrologic alteration^19^ and bioclimatic variables computed in the widely used WorldClim dataset^20^ as well as the CMCC-BioClimInd dataset^21^. Datasets of derived bioclimatic indicators have been proven essential for ecological applications in the terrestrial realm, notably for projecting potential climate change impacts on biodiversity^22,23^, but an equivalent for freshwater environments was lacking.

**Table 1:** Overview of output variables discharge (streamflow) and water temperature, available for each scenario and GCM (see Fig. 1, GCMs used are gfdl, hadgem, ipsl, miroc and noresm).

Variable	Scenarios	Forcing	10-year chunks
Discharge (streamflow, Q), weekly average [m^3^/s]	Historical (hist)	5 GCMs, E2O	1976 (1979) −1985, 1986–1995, 1996–2005
	Future: rcp2p6, rcp4p5, rcp6p0, rcp8p5	5 GCMs	2006–2019, 2020–2029, 2030–2039, 2040–2049, 2050–2059, 2060–2061, 2070–2079, 2080–2089, 2090–2099
Water temperature (WT), weekly average [K]	Historical (hist)	5 GCMs, E2O	1976 (1979) −1985, 1986–1995, 1996–2005
	Future: rcp2p6, rcp4p5, rcp6p0, rcp8p5	5 GCMs	2006–2019, 2020–2029, 2030–2039, 2040–2049, 2050–2059, 2060–2061, 2070–2079, 2080–2089, 2090–2099

Output is stored in 10-year chunks to keep file sizes manageable. Note that the historical simulation forced by E2O reanalysis data starts in 1979, the GCM-forced simulations start in 1976. Also, the first chunk of the future scenarios spans 14 years (2006 to 2019).

**Table 2:** Ecologically relevant derived variables (bioclimatic indicators) for streamflow Q.

Category	Variable	Code	Bioclim analogy
Magnitude	Annual mean streamflow	Q-mean	BIO12
	Minimum weekly flow	Q-min	—
	Maximum weekly flow	Q-max	—
	Mean flow of the wettest month	Q-wm	BIO13
	Mean flow of the driest month	Q-dm	BIO14
	Mean flow of the hottest month	Q-hm	—
	Mean flow of the coldest month	Q-cm	—
	Mean flow of the wettest quarter	Q-wq	BIO16
	Mean flow of the driest quarter	Q-dq	BIO17
	Mean flow of the hottest quarter	Q-hq	BIO18
	Mean flow of the coldest quarter	Q-cq	BIO19
Duration	Number of zero flow weeks	Q-zfw	—
Variability	Annual streamflow range	Q-range	—
	Streamflow seasonality index *σ(Q)/Q-mean*	Q-si	BIO15
	Baseflow index *Q*_90_*/Q-mean*	Q-bfi	—
	Hydrological variability index *(Q*_25_–*Q*_75_*)/Q*_50_	Q-hvi	—
Timing	Week of minimum weekly flow	Q-wmin	—
	Week of maximum weekly flow	Q-wmax	—
	Driest or wettest month	e.g. precipitation_wettest_month *	
	Driest or wettest quarter	e.g. precipitation_wettest_quarter*	

Categories are based on indicators of hydrologic alteration^19^. The bioclim-analogy (BIOXX) refers to the bioclimatic variables of the worldclim dataset^20^ and the CMCC-BioClimInd dataset^21^. These derived variables are available for each GCM-RCP combination, for 1976–2005 (1979–2005 for E2O); 2021–2040; 2041–2060; 2061–2080; 2081–2099. For details on how these variables were derived, see user notes and/or the scripts used (see Code Availability). The baseflow index and hydrological variability index,are provided for each year and are derived following Pastor *et al*.^30^.

**Table 3:** Ecologically relevant derived variables for water temperature (WT).

Category	Variable	Code	Bioclim analogy
Magnitude	Annual mean water temperature	WT-mean	BIO1
	Minimum weekly water temperature	WT-min	BIO6
	Maximum weekly water temperature	WT-max	BIO5
	Mean water temperature of the wettest month	WT-wm	BIO31
	Mean water temperature of the driest month	WT-dm	BIO30
	Mean water temperature of the hottest month	WT-hm	BIO28
	Mean water temperature of the coldest month	WT-cm	BIO29
	Mean water temperature of the wettest quarter	WT-wq	BIO8
	Mean water temperature of the driest quarter	WT-dq	BIO9
	Mean water temperature of the hottest quarter	WT-hq	BIO10
	Mean water temperature of the coldest quarter	WT-cq	BIO11
Duration	Number of weeks with WT =< 0.5 °C	WT-ztw	—
Variability	Annual water temperature range	WT-range	BIO7
	Water temperature seasonality index *σ(WT)/WT-mean*	WT-si	BIO4
Timing	Week of minimum water temperature	WT-wmin	
	Week of maximum water temperature	WT-wmax	
	Hottest or coldest month	e.g. air_temperature_ hottest_month*	
	Hottest or coldest quarter	e.g.air_temperature_coldest_quarter*	

Categories are based on indicators of hydrologic alteration^19^. The bioclim-analogy (BIOXX) refers to the bioclimatic variables of the worldclim dataset^20^ and the CMCC-BioClimInd dataset^21^. These derived variables are available for each GCM-RCP combination, for 1976–2005 (1979–2005 for E2O); 2021–2040; 2041–2060; 2061–2080; 2081–2099. For further details on how these variables are derived, see usage notes and/or the scripts used (included in the data records).

We produced the dataset using the state-of-the-art global hydrological model PCR-GLOBWB, validated by Sutanudjaja *et al*.^24^, coupled to the Dynamical Water temperature model DynWat, validated by Wanders *et al*.^13^. These are the only models currently capable of computing streamflow and water temperature globally at a native resolution of 5 arcminute (approximately 10 km at the equator). We forced the models with meteorological time series from five general circulation models (GCMs) selected by ISI-MIP^25^ (the Inter-Sectoral Impact Model Intercomparison Project) for the historical period as well as four climate scenarios (RCPs, Representative Concentration Pathways), thus covering a range of future climate scenarios. A historical simulation forced with reanalysis data is available as well. Streamflow and water temperature are available for the historical period as well as each RCP and each GCM in netCDF format^26^.

## Methods

### Workflow

**Fig. 1:**
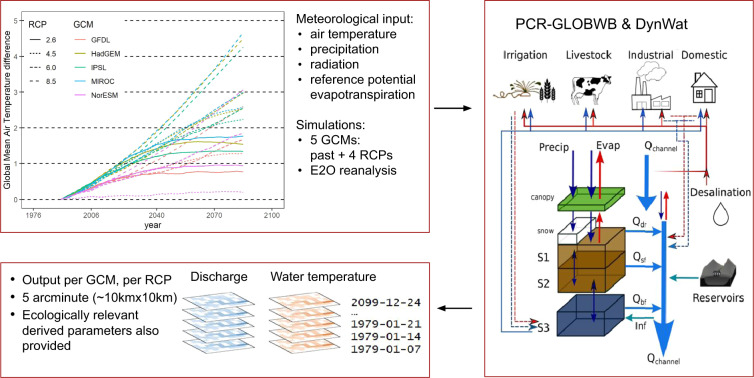
Schematic overview of the study design. The top left figure shows 30-year running mean global air temperature difference relative to 1976–2005 for each ISI-MIP GCM-RCP combination^25^. Temporally and spatially varying meteorological inputs are provided to PCR-GLOBWB and DynWat (right panel, from Sutanudjaja *et al*.^24^). The thin red lines indicate surface water withdrawal, the thin blue lines groundwater abstraction, the thin dashed lines return flows from water use. For DynWat see Wanders *et al*.^13^. The bottom-left panel shows the model outputs, which are weekly gridded discharge and water temperature per GCM, for the historical period and each RCP, at 5 arcminute resolution, as well as ecologically relevant derived variables.

We created the FutureStreams dataset using a combination of General Circulation Model (GCM) output, reanalysis data and state-of-the-art hydrological and water temperature models (Fig. 1). We obtained the GCM output from the Inter-Sectoral Impact Model Intercomparison Project (ISI-MIP) ensemble. The ISI-MIP ensemble consists of output from five CMIP5 GCMs, for four Representative Concentration Pathways (RCPs, thus 20 scenarios in total)^25^, which is downscaled to 0.5° and bias-corrected against the Watch Forcing Dataset^27^ (WFD). FutureStreams also includes a historical simulation forced with bias-corrected E2O (Earth2Observe) reanalysis data^28^. We used historical meteorological time series as well as future projections from the GCMs under the four RCP scenarios as input to the global hydrology and water resources model PCR-GLOBWB^24^. The hydrological model produces runoff that is used with the high-resolution water temperature model DynWat to simulate water temperature and streamflow time series. Both PCR-GLOBWB and DynWat run at a native resolution of 5 arcminute (approximately 10 km at the equator).

### Hydrological and water temperature models

The global hydrology and water resources model PCR-GLOBWB uses daily meteorological inputs to simulate the hydrological response in terms of local runoff. If the meteorological input data, such as the GCM input used here, has a lower spatial resolution than the 5 arcminute resolution of PCR-GLOBWB, the model statistically downscales the data to 5 arcminute based on spatial patterns in historical meteorological data^24^. From this, PCR-GLOBWB computes the water balance between two soil layers and a groundwater layer, and has up to four land cover types per grid cell^24^. Local runoff at the surface as well as in the soil and groundwater layers is routed along the river network using the kinematic wave approximation and includes floodplain inundation (Wanders *et al*. 2019). PCR-GLOBWB includes historical development of over 6,000 man-made reservoirs as well as water use for irrigation, industry, livestock and domestic purposes. Streamflow and meteorological inputs, such as air temperature and radiation, are then used to force DynWat to derive dynamic physically based water temperature estimates^13^. DynWat includes temperature advection, radiation and sensible heating, ice formation and breakup, thermal mixing and stratification in larger water bodies, as well as effects of water abstractions and reservoir operations at a spatial resolution of 5 arcminute with a daily global coverage.

### Historical and climate scenario forcing from ISI-MIP GCMs

We forced PCR-GLOBWB with output from the five ISI-MIP GCMs (GFDL-ESM2M, HadGEM2-ES, IPSL-CM5A-LR, MIROC-ESM-CHEM, NorESM1-M) for four RCPs (2.6, 4.5, 6.0 and 8.5 W/m^2^ in 2100). These GCMs were selected by ISI-MIP from the full CMIP5 ensemble to cover the full envelope of potential climate changes from wet to dry and warm to cold (Warszawski *et al*. 2013).

We used the GCM output to produce a baseline scenario for the historical period 1976–2005 and projections for the period 2006–2099. The GCM inputs are bias-corrected by ISI-MIP to correct the climate model data for systematic deviations of the simulated historical data from observations^27^, and are provided at a spatial resolution of 0.5° and a daily temporal resolution. The GCMs will therefore reproduce the correct statistical properties of floods and droughts (e.g. mean, severity, duration), but not necessarily the exact timing as observed in the historic record.

We obtained daily mean surface air temperature, precipitation, reference potential evapotranspiration and downward surface solar radiation from the ISI-MIP ensemble and used these to force the hydrological and water temperature models. As simulations of cloud cover and relative humidity are not bias-corrected via the ISI-MIP ensemble, we used CRU TS3.21 monthly climatology^29^.

### Historical reanalysis-forced simulation

We also provide streamflow and water temperature from a historical simulation forced by reanalysis data (1979–2005). Reanalysis data consists of observations assimilated into weather models, to create consistent and globally complete time series. A simulation forced with reanalysis data therefore enables validation of the model not only with respect to climatology and variability, but also with respect to timing of actual events such as droughts or floods. Output from a reanalysis-forced simulation can furthermore be used in comparison to the GCM-forced historical simulations to assess how well the GCM-forced simulations are capable of capturing the climatology and variability. Here we used the Earth2Observe (E2O) reanalysis data for a historical simulation from 1979 to 2005. E2O uses WFDEI data (WATCH forcing data methodology applied to ERA-Interim^28^).

### Simulations and output

We forced the PCR-GLOBWB model with the meteorological data from ISI-MIP and E2O as described above. We started the simulations in 1951 with initial conditions from an earlier E2O-forced simulation. Subsequent years (1951–1975) are considered spin-up. The E2O reanalysis-forced simulation starts in 1976 directly from the initial conditions.

We performed the simulations at SurfSara Cartesius, the national e-infrastructure for Dutch universities and institutes, parallelized the calculations along watershed boundaries^24^, and aggregated the output using Python. From the model output, we extracted weekly streamflow and temperature values for each year from 1976 through 2099. The output is grouped in 10-year chunks, separately for each GCM and RCP (Table 1^26^). In addition, we calculated a set of derived streamflow and temperature indicators that are expected to be particularly relevant from an ecological perspective (Tables 2 and 3). We calculated these derived variables as long-term aggregates/averages for six periods aligned with those commonly used in the worldclim dataset^20^ as well as the CMCC-BioClimInd dataset^21^: 1976–2005 (1979–2005 for E2O); 2021–2040; 2041–2060; 2061–2080; 2081–2099. These derived variables are also provided through^26^.

## Data Records

Available data files at Yoda^26^:

- weekly streamflow (m^3^/s, called discharge in filename) and water temperature (K) globally from 1976 through 2005 for the historical period, or 1979–2005 for the E2O simulation, and 2006 through 2099 for each RCP (2.6, 4.5, 6.0 and 8.5), stored in chunks of 10 years per GCM (GFDL - HadGEM - IPSL - MIROC - NorESM), see Table 1.
- set of ecologically-relevant (indicator) variables derived from the weekly values: see Tables 2 and 3, as well as Usage notes below and scripts (Code Availability) for more details.
- masks (see Usage notes below)

The netCDF4 files have regular latitude - longitude grids with a cell size of 5 arcminute (~10 km) and a global extent, including all continents except for Antarctica (90°N to 90°S latitude and 180°W to 180°E longitude).

## Technical Validation

### Water temperature records

Wanders *et al*.^13^ have validated water temperatures from DynWat, using the same model set-up and the ERA-40 and ERA-Interim reanalysis data as forcing. They showed that the modeled temperature matches observed temperatures well (R^2^ = 0.861, using observations at 358 locations), and that DynWat is capable of capturing spatial patterns and trends in water temperature, thereby providing confidence in the quality of the dataset.

### Streamflow records

Sutanudjaja *et al*.^24^ showed that seasonality, inter-annual anomalies, and the general discharge characteristics in PCR-GLOBWB compare well to observations, especially at the 5 arcminute resolution (modeled discharge compared to time series at 5,363 locations gives an R^2^ mode between 0.7 and 0.8). They furthermore showed that PCR-GLOBWB is able to reproduce trends and seasonality in total water storage, as observed by satellite measurements.

## Usage Notes

### Variable names, units and timestamps

Streamflow is runoff routed along a drainage network, in m^3^/s, also known as discharge, which is the variable name used in the files. Water temperature is given in units of Kelvin. Filenames include the variable name, GCM, scenario (hist for historical, or one of the RCPs) and the time period (years). The timestamps in the files reflect the last date of the period over which the output was averaged, so the first timestamp of the weekly averages is January 7th 1976.

### Ecologically-relevant variables

The ecologically-relevant streamflow and water temperature variables derived from the weekly values are established based on a combination of classification frameworks, i.e., indicators of hydrologic alteration^19^, terrestrial bioclimatic variables in the worldclim dataset^20^ as well as the CMCC-BioClimInd dataset^21^, aggregated accordingly: 1976–2005 (1979–2005 for E2O); 2021–2040; 2041–2060; 2061–2080; 2081–2099. The scripts used to compute these derived variables can be found under Code Availability.

For files containing information on timing (see Tables 2–3), note that the counting is 0-indexed. So week numbers run from 0 through 51, months from 0 to 11. For timing of quarters, 0 is DJF, 1 is MAM, 2 is JJA, 3 is SON. The week number (for WT-wmin, WT-wmax, Q-wmin, Q-wmax) is determined as the mode, i.e. the most frequent week number within a period. For each period (20, 25 or 30 years) we looked for the week number in which the minimum or maximum water temperature or discharge occurs. If that happens most often in week X, that week number is stored. It can however occur that a certain minimum/maximum temperature or discharge occurs equally often in multiple weeks - then we assign a missing value.

The variables Q-bfi and Q-vi are calculated according to Pastor *et al*.^30^. The baseflow index is an indicator of the importance of stored sources; a high index indicates that flow is mostly sustained by stored sources such as groundwater.

Scripts used to create the derived variables are available through the FutureStreams GitHub repository (see Code Availability below).

### Multi-model set-up

We provide future scenarios for four RCPs (representative concentration pathways; 2.6, 4.5, 6.0 and 8.5 W/m^2^ in 2100) for the five ISI-MIP GCMs. Projections differ across RCPs due to differences in greenhouse gas forcing, and across GCMs due to differences in e.g model parameterization and resolution. Generally the spread across GCMs is larger than that across RCPs^7,31^. When interested in the general effect of climate change, users are advised to use the mean or median across the GCMs, rather than selecting a specific GCM. When interested in the spread across GCMs, users can explore or represent that in various ways, such as color intensity indicating agreement amongst models^5^, bar or violin plots^7^ etc.

### Warming levels

To facilitate assessments and comparisons of streamflow and water temperature at a certain air temperature rise rather than specific years^5,7^, we provide a table with the years in which each GCM/RCP reaches the global mean temperature rises 1.5°, 2.0°, 3.2°, 4.5° compared to pre-industrial temperatures (as used by Barbarossa *et al*.^7^) with our scripts (see Code Availability). These years represent the central value of a 30-year running mean, so users should evaluate the 30-year mean (or other statistic) of discharge or water temperature centered around the year that a certain warming level is reached, which is specific to each RCP and GCM combination. For instance, if 1.5° warming is reached in 2040, the 30-year period 2025–2054 should be considered.

### GCMs, bias-correction and reanalysis data

The majority of our simulations are forced with meteorological time series from GCMs. Those are bias-corrected^27^ before being applied to impact models such as PCR-GLOBWB, which corrects for systematic deviations of the simulated historical data from observations. For instance, for temperature the offset in average temperature in the historical GCM simulation with respect to observations is subtracted from temperatures in all scenarios of that GCM. The bias-corrected GCM forcing should thus well represent climatology, but not necessarily timing of actual events such as floods and droughts. Reanalysis data is created by assimilating observations into weather models, to obtain consistent and globally complete time series. The output of the simulation forced with meteorological time series from the (E2O) reanalysis data should therefore reflect not only the average streamflow and water temperatures, but also timing of actual events such as droughts.

If users want to check for themselves how the GCM-forced historical simulations discussed here deviate from reanalysis-forced simulations, they can use the output from the E2O-forced simulation provided here, the monthly output linked to Wanders *et al*.^13^ (see also Code Availability) or the daily output of those simulations which are available from Niko Wanders upon request. The latter are forced with ERA-40/ERA-Interim reanalysis data.

### Notes of caution

Beware of temperature in grid cells where streamflow is low, which can cause temperatures to become unrealistically high due to strong fluctuations in the water level. The computational timesteps currently implemented in DynWat are not sufficiently small to provide stable solutions for these conditions. For some lakes and reservoirs we observe a similar problem when lakes expand or shrink as a result of water levels changes. These locations can be masked and we can assume that water temperature follows the air temperature for these very shallow water layers. A file with locations of lakes and reservoirs is provided in the data repository (under indicators/mask) so users can mask these if desired.

Furthermore, we provide masks for each GCM-RCP-period which users can apply to the derived variables if desired. These masks are based on Q-mean and WT-mean and thresholds of 10 m^3^/s and 350 K, respectively. They can be found in the data repository (i.e. indicators/waterTemperature/WT-mask). The scripts used to create these masks are provided through the FutureStreams GitHub repository (see Code Availability below), which can be used to create masks with different thresholds. These scripts are called mask_unrealistic_values.py and maskFunctions.py.

We also provide scripts to mask out unrealistic values directly in the weekly Q and WT files, these scripts are mask_unrealistic_values_weekly.py and maskFunctions_weekly.py. In all these scripts the threshold for discharge is set to 10 m3/s and for water temperature to 350 K, but users can change those to their preferred values. The threshold value will be included in the resulting output file name.

**Fig. 2:**
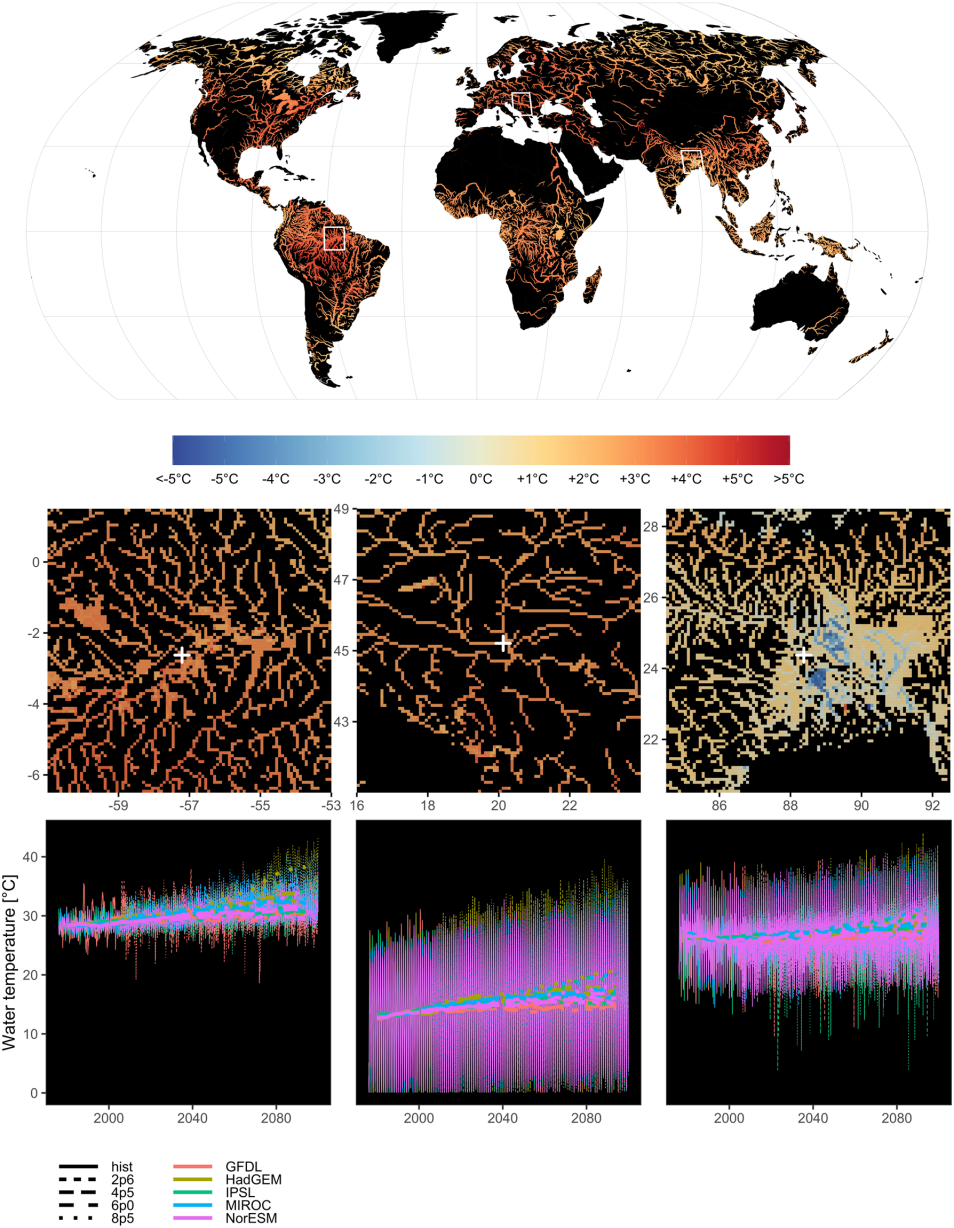
Water temperature [°C] anomaly. The maps show the difference between the mean water temperature over the period 2070–2099 (RCP8p5) and the historical period 1975–2005. The map shows values only for rivers with streamflow greater than 50 m^3^/s and the width of the rivers is scaled based on the streamflow values for clarity of representation. Insets below the map show the original gridded resolution at 5 arcminute for cells with streamflow values greater than 10 m^3^/s. The bottom insets show water temperature time series sampled at specific grid-cell locations (white crosses in the insets) for the Amazon (−57.2083° longitude, −2.625° latitude), Danube (20.125° lon, 45.2083° lat) and Ganges (88.375° lon, 24.375° lat). Time series are represented for each GCM and RCP available within FutureStreams; thin lines represent weekly values, thick lines represent 10 year rolling means.

Furthermore, we encountered spin-up issues in some pixels for the future RCP simulations. Instead of following the temperatures from the end of the historical simulation, temperatures drop at the beginning of the future simulation, so the first few weeks of 2006 temperatures can be unrealistically low. In Fig. 2, output of the year 2007 is used for the year 2006 .

**Fig. 3:**
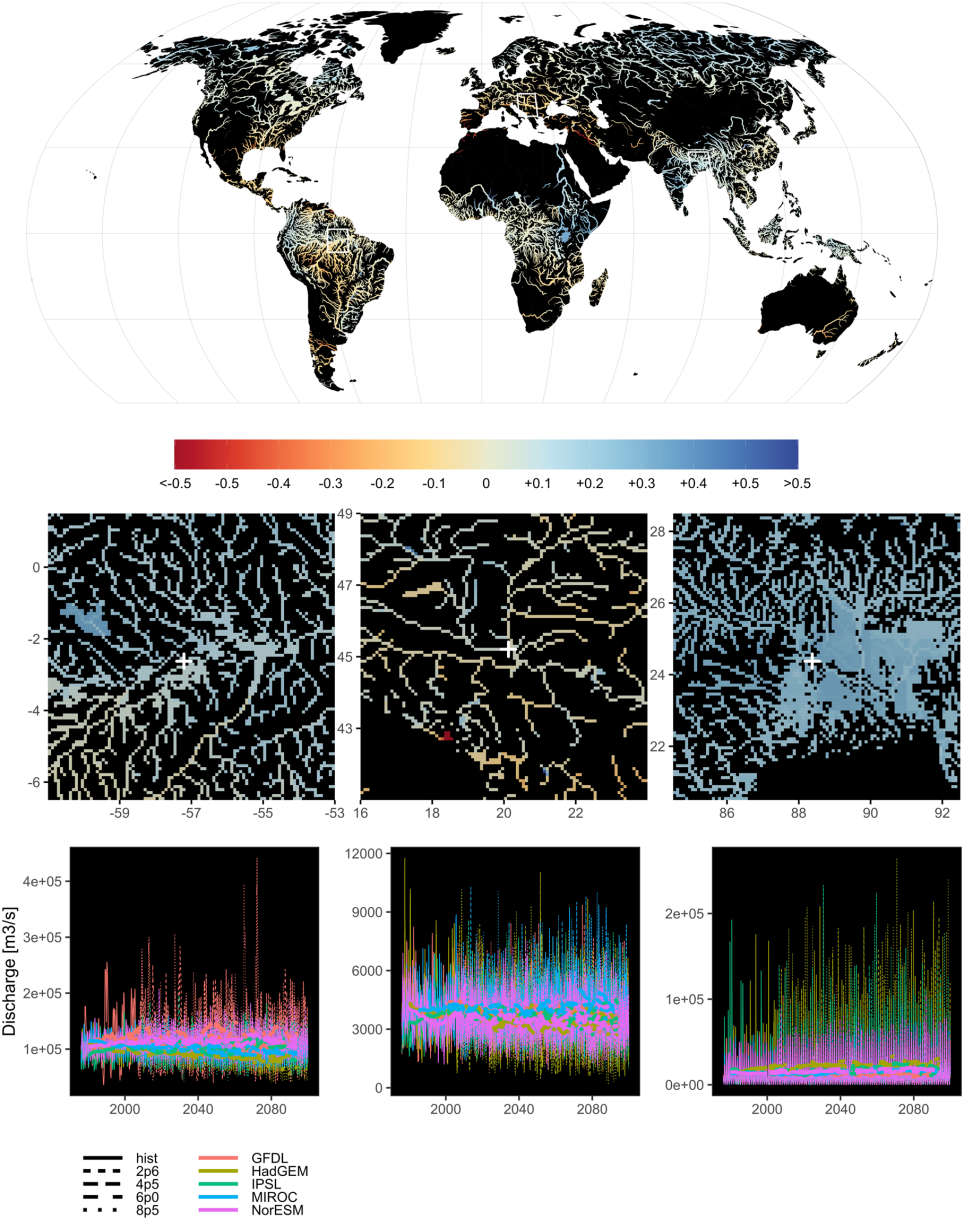
Streamflow [m^3^/s] anomaly. The maps show the difference between the log10 transformed mean streamflow over the period 2070–2099 (RCP8p5) and the log10 transformed mean streamflow over historical period 1975–2005. The map shows values only for rivers with streamflow values greater than 50 m^3^/s and the width of the rivers is scaled based on the streamflow values for clarity of representation. Insets below the map show the original gridded resolution at 5 arcminute for cells with streamflow values greater than 10 m^3^/s. The bottom insets show water temperature time series sampled at specific grid-cell locations (white crosses in the insets) for the Amazon (−57.2083° longitude, −2.625° latitude), Danube (20.125° lon, 45.2083° lat) and Ganges (88.375° lon, 24.375° lat). Time series are represented for each GCM and RCP available within FutureStreams; thin lines represent weekly values and thick lines represent 10 year rolling means.

**Fig. 4:**
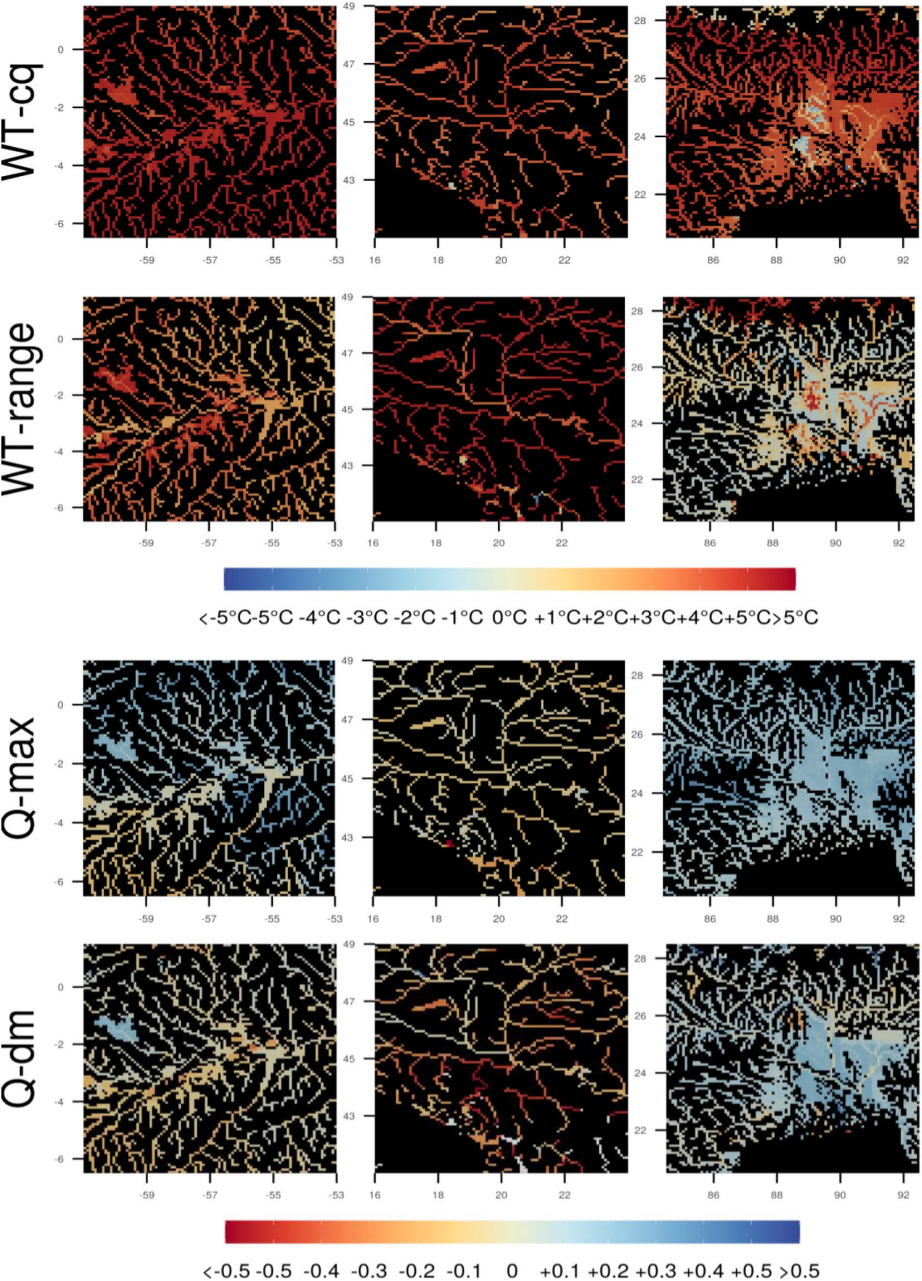
Anomalies for selected ecologically relevant derived variables (bioclimatic indicators) for the same areas in the Amazone (left), Danube (middle) and Ganges (right) basins as used in Figs. 2 and 3. Differences are shown between RCP8.5 2080–2099 and 1976–2005. WT-cq is the water temperature of the coldest quarter, WT-range is temperature range, Q-max is maximum streamflow, Q-dm is streamflow of the driest month (see also Tables 2 and 3 below). For streamflow we show the difference between log10-transformed flow.

## Data Availability

Weekly water temperature and flow estimates at 5 arcminute were created using PCR-GLOBWB and DynWat. The PCR-GLOBWB model code is available at https://github.com/UU-Hydro/PCR-GLOBWB_model as well as 10.5281/zenodo.247139^32^, and the global input files are available through 10.5281/zenodo.1045339^33^. The DynWat code is available via https://github.com/wande001/dynWat. Monthly output from Wanders *et al*.^13^ is available through 10.5281/zenodo.3337659^34^. Scripts used for figures in this paper are available through https://github.com/vbarbarossa/futurestreams_figures. Lastly, the repository https://github.com/JoyceBosmans/FutureStreams contains the scripts used to create the ecologically relevant derived variables, scripts to mask out grid cells with unrealistic values (see User Notes above), the script used to create Fig. 4, an example configuration file for PCR-GLOBWB as well as a table with years in which warming levels are reached for each RCP and GCM combination. All the model runs were carried out on the Dutch national e-infrastructure Cartesius.
